# The Elevation of *IRSp53* Expressing Level in Colon Cancer Specimens and the Secretome of hAMSCs' Therapeutic Impacts on Tumor Growth Promotion via Inhibiting of EGFR/c-Src/IRSp53/p-AKT/p-Stat3/cyclin D1 Signaling Cascade in HT-29 Colon Cancerous Cell Line

**DOI:** 10.1155/sci/7434211

**Published:** 2025-10-29

**Authors:** Mana Alavi, Fatemeh Safari, Saba Fakhrieh Asl, Fariborz Mansour-Ghanaei

**Affiliations:** ^1^Department of Biology, Faculty of Science, University of Guilan, Rasht, Iran; ^2^Gastrointestinal and Liver Disease Research Center, Guilan University of Medical Science, Rasht, Iran; ^3^Caspian Digestive Diseases Research Center, Guilan University of Medical Science, Rasht, Iran

**Keywords:** cancer therapy, 3D cell culturing technique, EGFR/c-Src/IRSp53/p-AKT/p-Stat3/cyclin D1 signaling pathway, HAMSCs, HT-29 colon cancerous cells

## Abstract

Cancer is a predominant testimony of human departure in a global way. Current therapeutic strategies are not sufficient, and thereby exploring a new approach with high efficacy and influence is desired. The intention of this research is to distinguish a novel therapeutic burgeon in colon cancer plus employing the human mesenchymal stem cells (hAMSCs) secretome as a new tool in colon cancer therapy. For this purpose, 30 pieces from patients afflicted with colon cancer were provided. The expressing level of IRSp53 was evaluated using quantitative real-time PCR (qRT-PCR). Then, a coculture procedure utilizing six well plates transwell was applied. Since 72 h, tumor increment was surveyed in HT-29 cells treated by hAMSCs through the EGFR/c-Src/IRSp53/p-AKT/p-Stat3/cyclin D1 signaling cascade. Our results indicated *IRSp53* upregulation in patients suffering colon cancer and reduction of EGFR/c-Src/IRSp53/p-AKT/p-Stat3/cyclin D1 signaling pathway, which led to suppression of cell proliferation in the hAMSCs-treated HT-29 colon cancerous cells. We also found tumor growth suppression as well as IRSp53 expression in hAMSCs-treated HT-29 colon cancerous cell line using a 3D cell culturing technique. Our study's findings indicate that colon cancer therapy could benefit from targeting IRSp53 and that MSCs could be a valuable therapeutic option for stopping the proliferation of colon cancer cells. This could be achieved through the EGFR/c-Src/IRSp53/p-AKT/p-Stat3/cyclin D1 signaling pathway.

## 1. Introduction

Colon cancer is responsible for a significant number of cancer-related fatalities globally, ranking fifth among various types of cancer [1]. Surgery and chemotherapy are currently considered the major therapeutic approaches. It appears that current strategies are not efficient enough, and therefore, identification of new methods with higher efficiency and fewer side effects is desired. Cancer therapy based on mesenchymal stem cells (MSCs) is currently well established [2, 3]. Along with their unrivaled peculiarities, as well as straightforward in vitro isolating and culture, it has been reckoned that MSCs could be a highly potential facility in cancer remedy [4, 5].

The insulin receptor substrate of 53 kDa, or IRSp53 (also known as BAIAP2) is an adaptor protein in the inverse BAR (I-BAR) domain-containing protein subfamily. The members of this subfamily have been reported to modulate motility of cells and membrane deformation through actin polymerization and plasma membrane protrusion formation [6]. The role of IRSp53 in tumorigenesis is still not clear. It was reported that IRSp53 is tyrosine phosphorylated at multiple sites in reacting to epidermal growth factor receptor (EGFR) stimulation [7]. Furthermore, it was found that the extenuation of IRSp53 alleviated the proliferating of cells in v-Src-transformed cells (IV5) and tumor formation in mice. Additionally, knocking down of IRSp53 decreased the expression level of AKT (by inhibition of phosphorylation at the Ser473 site) and Stat3 (by inhibition of phosphorylation at Tyr705 site) and decreased cyclin D1 expression, thus facilitating the arrest of cellular cycle progression [8]. IRSp53 is a substrate in EGFR signaling pathway. Src known as a pivotal intercessor in signaling cascades related to EGFR. Moreover, the elevation of Src activity in gastrointestinal cancers was previously demonstrated. In this regard, the overexpression of Src in approximately 80% of colorectal cancer (CRC) specimens had been reported [9–12]. Also, high expression levels of EGFR in CRC were previously displayed [10]. Hence, the suppression of EGFR and Src is one of the most significant targets in cancer treatment [13]. We previously found that MSCs secretome is able to suppress not only EGFR but also Src expressing levels and activity in a variety of cancerous cells [14, 15].

In the present investigation, we concentrated on colon cancer to evaluate the expression of *IRSp53* by utilizing quantitative real-time PCR (qRT-PCR). Moreover, we administered the coculturing method employing a 6-well plate transwell. Following 72 h, enhancement of tumor formation was appraised in human MSCs (hAMSCs)-treated HT-29 cells by EGFR/c-Src/IRSp53/p-AKT/p-Stat3 signal network employing qRT-PCR, besides the Western blot technique, as well as the MTT and hanging drop assays. The outcomes of our study indicate that MSCs could potentially serve as an efficient treatment strategy for halting the proliferating of colon cancerous cells via the EGFR/c-Src/IRSp53/p-AKT/p-Stat3/cyclin D1 signaling transduction process.

## 2. Methods and Materials

### 2.1. Sample Purveyance

Thirty samples were gathered from both tumor mass and adjacent noncancerous tissues (13 men as well as 17 women, range of age: 36–89 years; in Gastrointestinal and Liver Disease Research Center and Caspian Digestive Diseases Research Center, Rasht, Iran). Cancer staging was defined under the American Joint Committee on Cancer Staging (AJCC, stage I: 16.6%; stage II: *n* = 40%; stage III: 26.6%; stage IV: 16.6%). The patients did not receive radiotherapy or chemotherapy. All patients provided written informed consent. Tumor specimens were stored at −80˚C.

### 2.2. Line of Cells and Circumstances of Culture

HAMSCs afforded by the Iranian Biological Resource Center (IBRC, Cat No: C10893, male, newborn, fibroblast-like), but all colon cancerous (HT-29), pancreatic cancerous (MiaPaca2), as well as breast cancerous (MDA-MB-231) and prostate cancerous (LNCaP) cell lines were procured from the Pasteur Institute (Tehran, Iran). The MSCs recognition was confirmed due to being negative for CD45 as well as CD33, but positive for not only CD105 but also CD90 and CD73. The cellular culture conditions were similar to our previous reports and studies [14].

### 2.3. Structure of Coculture (HT-29 and MSCs)

In the experiment, 150,000 HT-29 colon cancerous cells initially were placed and grew on the lower area of a six-well plate. Within the next day, MSCs were implanted at an equal density (1:1 ratio of MSCs to HT-29 cells) on the uppermost section of a polycarbonated transmembrane filter in a transwell filter style (BD Falcon, Bedford, MA, USA) by a pore size of 0.4 μm, as per similar previous studies. Beyond 72 h, for analyzing both the control and remedied HT-29 cancer cell lines were utilized for RNA extraction besides Western blot [14].

### 2.4. RNA Extracting, cDNA Production Plus qRT-PCR

Upon72h, first, HT-29 cancerous cells, which were treated via hAMSCs, were aggregated, then undergone lysing and recruited for qRT-PCR tests. The status of qRT-PCR was formerly related. The exploited primers of *GAPDH*-F: 5′-CAA GGT CAT CCA TGA CAA CTTTG-3′, R: 5′-GTCCACCACCCTGTTGCTGTAG-3′; *IRSp53*-F: 5′-GCCCAAATCCCTGTCTCCTC-3′, R: 5′- CTCGGTGGTGGCATAGCTG -3′ [14]. The PCR was subjected to thermal conditions that included an initial denaturating at 94°C for 3 min, followed by 35 cycles of denaturing at 94°C for 35 s, annealing at 63°C for 45 s, and amplifying at 72°C for 45 s. The repetition number of the entire experiment was three. Furthermore, the primers were provided by Pishgam Biotech Co., (Tehran, Iran).

### 2.5. Cellular Viability Trial

3-(4, 5-dimethylthiazol-2-yl)−2, 5-diphenyl tetrazolium bromide (MTT) assay was exercised in order to explore how the HT-29 colon cancerous cells' viability was influenced by the secretome of hAMSCs. Briefly, HT-29 cancer cells were placed in 96-well culture plates (5 × 10^3^ cells/well). After 24 h incubation, the cells were treated with hAMSCs secretome. After 48 h, the medium was removed, and 100 μL of MTT reagent (1 mg/mL) was added to each well, and cells were further incubated at 37°C for 4 h. The MTT solution was removed, 50 μL of DMSO was added to each well to dissolve formazan crystals, and the plates were gently shaken for 10 min, followed by reading by an ELISA plate reader (BiotekELx 800, USA) [14].

### 2.6. Protein Expressing Investigation

Anti-B-actin (C4: sc-47778), anti-EGFR (A10, sc373746), anti-c-Src (B-12: sc-8056), anti-p27 (F-8: sc-1641), anti-IRSp53 (46: sc-136470), anti-Stat3 (F-2: sc-8019), anti-AKT (B-1; sc-5298), and anti-cyclin D1 (A-12; sc-8396) supplied by Santa Cruz Biotechnology. Anti-p-Stat3 (Tyr 705; No: 651001) and p-AKT (Ser 473; P31751) were obtained from BIOLEGEND and Cell Signaling, respectively. In brief, HT-29 cancer cells were harvested and lysed in lysis buffer (50 mM Tris -HCl, pH 7.5, 150 mM NaCl, 1 mM EDTA, 1% Triton X-100, 2 mM Na3VO4, 1 mM PMSF). Total cell lysates (TCLs) were subjected to SDS-PAGE. Proteins transferred to PVDF membrane filters (Millipore) were soaked in solutions containing primary antibody (1:300, 90 min at room temperature) and secondary antibody (1:1000, 45 min at room temperature). Western blot chemiluminescence reagent (PerkinElmer Life Sciences) was applied to visualize the bands. Intensities of chemiluminescence on the immunoblotted filters were quantitated by using a luminescence image analyzer (LAS-4000, Fuji Film) [14].

### 2.7. Hanging Drop Constitution

To obtain a 3D cell culture model and form spheroids, the hanging drop technique was implemented. In essence, the HT-29 cells were grown, trypsinized, and quantified. The process involved pipetting 10 drops of 20 μL each, with 20 × 10^3^ cells in each drop, onto the lid of a 60 mm tissue culturing dish. After that, 5 mL of PBS was added to the bottom of the dish. Also, the controls (cells + medium) as well as samples (cells + stem cell-conditioned medium) underwent the same investigation. Additionally, the applied media was replaced every other day, as well as the size and quantity of spheroids, which were measured after they had formed, which typically took about 3 days. Eventually, the formation of a spheroid was assessed by engaging a phase-contrast inverted microscope (INV100, BEL Engineering, Italy) [14].

### 2.8. Actuarial Data Analysis

Through recruiting SPSS 22 (Chicago, IL, USA) besides Graph Pad Prism 7 software, the findings were deliberated and designed based on former experiments. Briefly, the data were expressed as mean ± standard deviation. Furthermore, the experiments were performed three times, and the groups were compared using an independent samples *t*-test. Finally, a *p*-value less than 0.05 was considered statistically significant [14].

## 3. Results

### 3.1. Upregulation of *IRSp53* in Colon Cancer Samples

**Figure 1:**
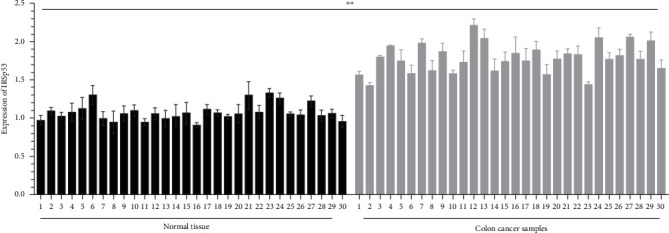
30 specimens from colon cancer patients (and 30 specimens from adjacent normal tissues as control) were collected. Relative expression of *IRSp53* mRNA was analyzed using qRT-PCR. The data represent mean ± SD of three independent experiments. ⁣^*∗∗*^*p*  < 0.0001 was considered to be statistically significant.

To determine the expressing level of *IRSp53* in colon cancer, a gathering of 30 pieces from the tumor mass and adjacent nontumorigenic tissues (13 men as well as 17 women, range of age: 36–89 years old) was carried out. The expression of *IRSp53* was detected through employing qRT-PCR (Figure 1). Upregulation of *IRSp53* expressing in colon cancerous experimented specimens was discovered in our investigations. It should note that the expression of IRSp53 in colon cancer tissues had no correlation with gender, age, stages of tumors, and pathological type.

### 3.2. Downregulation of IRSp53 in hAMSCs-Treated HT-29 Colon Cancerous Cells

**Figure 2:**
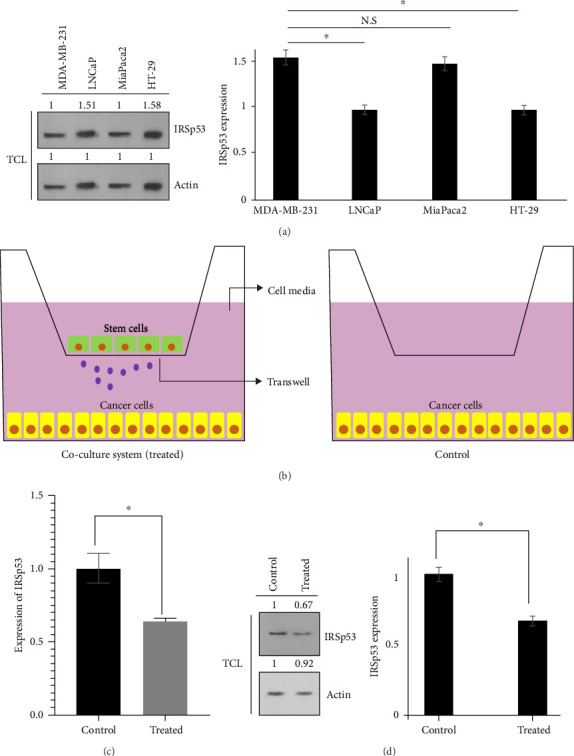
In different cancer cells, the expression of IRSp53 protein was detected using Western blot (A). Actin used as an internal control (TCL: total cell lysate). Schematic model of coculture system used in this study (B). The relative expression of IRSp53 in both gene and protein levels was shown (C, D). The data represent mean ± SD of three independent experiments. *⁣*^*∗*^*p*  < 0.05 was considered to be statistically significant. N.S, nonsignificant.

Out of IRSp53 overexpressed levels in colon cancerous specimens, the expression of IRSp53 in a number of cancerous cells, including LNCaP prostate, MiaPaca2 pancreatic, MDA-MB-231 breast and HT-29 colon cancerous cell lines, was deliberated in our experiment (Figure 2A). It was noticed that the levels of IRSp53 expression were elevated in HT-29 colon cancerous cells. As a result, we decided to focus on studying HT-29 colon cancerous cells further. Afterward, HT-29 colon cancerous cells underwent coculturing with hAMSCs cells, exploiting a transwell filter method (Figure 2B). Following 72 h, HT-29 colon cancerous cells that were treated by hAMSCs were collected in order to consider IRSp53 expression in not only a gene manner but also protein (Figure 2C, D). According to our findings, the expression of IRSp53 in HT-29 colon cancerous cells was found to be reduced as a result of being treated with hAMSCs.

### 3.3. The Reduction of Cellular Proliferation via Downregulating of EGFR/c-Src/p-AKT/p-Stat3/Cyclin D1 Signaling Network in HT-29 Cancerous Cells Treated by hAMSCs

**Figure 3:**
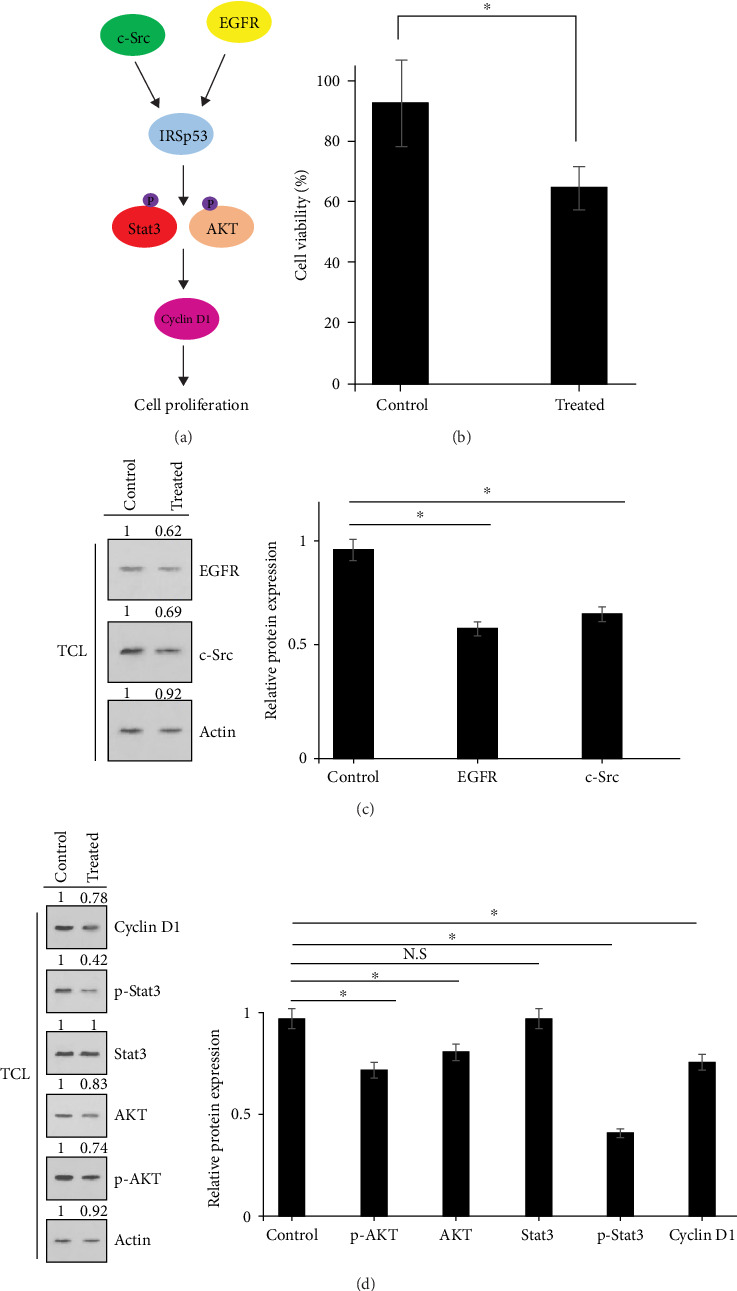
Schematic model of EGFR/c-Src/IRSp53/p-AKT/p-Stat3/Cyclin D1 signaling pathway used in this study (A). The inhibitory effect of hAMSCs secretome on HT-29 colon cancer cells using MTT assay. The data represent mean ± SD of three independent experiments. *⁣*^*∗*^*p*  < 0.05 was considered to be statistically significant (B). The expression of EGFR, c-Src, p-AKT, p-Stat3, and cyclin D1 proteins were shown using Western blot in hAMSCs-treated HT-29 cells (C, D). Actin used as an internal control (TCL: total cell lysate). N.S, Nonsignificant.

It has been found that IRSp53 is tyrosine phosphorylated at multiple sites in response to EGFR stimulation, and knocking down of IRSp53 diminished cell proliferation in cells that v-Src transformed and tumor formation in mice through p-AKT/p-Stat3/cyclin D1 signaling pathway (Figure 3A) [7, 8]. For evaluating possible impacts of the secretome of the hAMSCs on tumor growth plus progress via EGFR/c-Src/IRSp53/p-AKT/p-Stat3/cyclin D1 signaling pathway, first coculturing of HT-29 colon cancerous cells /hAMSCs was fulfilled for 72 h afterward, they underwent MTT assay (Figure 3B). Moreover, TCLs of HT-29 colon cancerous cells were provided thereupon recruited for Western blot (Figure 3C, D). These evidences displayed a suppressor role for secretome of hAMSCs in cellular proliferation by downregulating the EGFR/c-Src/p-AKT/p-Stat3/cyclin D1 signal transduction network in HT-29 colon cancerous cells.

### 3.4. Secretome of hAMSCs Suppressed Tumor Growth Promotion via Inhibiting of IRSp53 Expression Exploiting 3D Cellular Culturing Method

**Figure 4:**
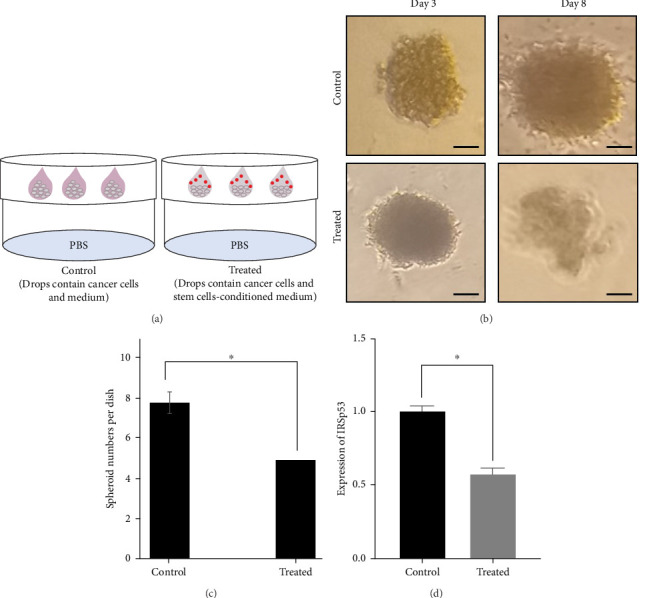
Schematic model of spheroid formation using the hanging drop technique (A). The size and number of spheroids under treatment with stem cell conditioned medium were shown (Magnification: 20x, scale bar: 100 μm; Three independent experiments were done. *⁣*^*∗*^*p*  < 0.05 was considered to be statistically significant) (B, C). The expression of *IRSp53* was shown using qRT-PCR in hAMSCs-treated HT-29 cells (D).

A 3D cellular culturing system was developed to evaluate a condition that is an indicator of cellular in vivo behaviors, and hence, the hanging drop system was administered [14, 15]. As previously described, upon passing of approximately 3 days, spheroids became manifest (Figure 4A). Then, we measured not only the size, but also the number of spheroids. As shown in Figure 4B, C, both the size and number of spheroids in the treated piece (consisting of cells and stem cell-conditioned medium) were lower than the control (consisting of cells and medium). Furthermore, IRSp53 expression level was mitigated in treated cells (Figure 4D). The 3D cell culturing method's outcome demonstrated that the secretome of the hAMSCs affects HT-29 cancerous cells to develop and progress in a therapeutical manner.

## 4. Discussion

Colon cancer is a type of cancer with high death rates worldwide. It seems that the current therapeutic strategies are not sufficient, and thus, exploring new methods with high efficiency and low side effects is a striking obstacle for interested researchers. Herein, we focused on colon cancer to figure out the remedial impacts related to the secretome of MSCs (as a new strategy) through EGFR/c-Src/IRSp53/p-AKT/p-Stat3/cyclin D1 signaling pathway. We found that the expression of *IRSp53* was elevated in the people who suffer from colon cancer. Moreover, the inhibitory impacts of the secretome of hAMSCs via EGFR/c-Src/IRSp53/p-AKT/p-Stat3/cyclin D1 signaling pathway were displayed in HT-29 cancerous cells.

In our former investigations, we found that IRTKS and Pinkbar (the other members of I-BAR domain-containing protein subfamily) may be considered as potential targets in cancer treatment using hAMSCs secretome [15, 16]. Due to the highly homologous structure of IRTKS and Pinkbar with IRSp53, we are interested in evaluating the possible role of IRSp53 in cancer therapy via hAMSCs secretome. It is worth noting that the exact role of IRSp53 in carcinogenesis is not well recognized yet [6]. In this respect, it has been indicated that the reduction of IRSp53 expression suppresses cell-related proliferating in cells that v-Src transformed as well as tumor formation in mice, and IRSp53 knockdown suppresses p-AKT/p-Stat3/cyclin D1 signaling pathway [8]. In Heung et al's. [7] research, it has been reported that IRSp53 was tyrosine phosphorylated at multiple sites in response to EGFR stimulation. It appears that activation of Src (or EGFR) upregulates IRSp53/p-AKT/p-Stat3/cyclin D1 signaling pathway. Our results indicated that IRSp53 is a substantial aim in colon cancer remedy and secretome of hAMSCs suppresses IRSp53 expression as well as related molecules such as IRSp53, p-AKT (Ser 473), p-Stat3 (Tyr 705), and cyclin D1 in the HT-29 colon cancerous cell line. Moreover, we displayed that IRSp53 expression of spheroids (based on a 3D cell culture model) decreased during treatment with MSCs secretome. Interestingly, we detected *IRSp53* expression in 30 specimens from colon cancer patients. There is no report about *IRSp53* expression in specimens from cancer patients, and therefore, our results indicate that IRSp53 may be presumed as an important gene/protein in colon cancer remedies.

Interestingly, Antoine et al. [17] showed interaction of SHIP2 with IRSp53 and Mena participates in the formation of multiprotein complexes to regulate membrane dynamics in MDA-MB-231 cancer cells. In another study, it was indicated that 14–3–3 binding to IRSp53 inhibits cancer cell chemotaxis [18]. Furthermore, it was shown IRSp53 could be involved in tumor cell growth via extracellular microvesicle secretion [19]. Taken together, it seems the mentioned molecules (that enable them to interact with IRSp53) could be critical targets in cancer therapy by MSCs secretome.

MSCs have remarkable features such as high in vitro self-renewal capacity and immunomodulatory characteristics that enable them to be a sustainable supply for therapeutic applications. Moreover, MSCs are able to differentiate into different cells, which is critical for their regenerative properties and facilitates tissue repair and regeneration [20, 21]. The MSCs secretome contains the vesicular fraction (such as microvesicles, exosome, and apoptotic bodies) and the soluble fraction (including cytokines, proteins, and trophic factors), and the balance between them is important for final paracrine impacts related to the secretome of MSCs on cancerous cells. The soluble fraction harbors factors with proinflammatory and anti-inflammatory properties, and the vesicular fraction transports nucleic acids and small proteins. These vesicles engage with target cells by binding to cellular receptors or delivering their cargo into the cell cytoplasm, triggering processes like angiogenesis, neurite outgrowth, and inflammatory responses [22, 23].

It was previously found that MSCs secretome has a dual effect on tumorigenesis (as a suppressor or inducer of tumor growth) depending on cell context [24–27]. Nowadays, MSCs secretome is supposed to be a neoteric potential procedure in treating various diseases, including uveitis, acute hepatic failure, spinal cord injury, and wound healing [28–31]. MSCs secretome components are not completely understood. As a future study direction, it is recommended to evaluate components of MSCs secretome separately. Moreover, expression of IRSp53 in different CRCs and the effects of MSCs secretome on CRCs should be analyzed.

Our study had several limitations: we analyzed numerous target proteins that have interaction with IRSp53 after treating HT-29 cancer cells with the secretome. However, more colon cancer cell lines (or different cancer cell lines) should be investigated. Also, in vivo experiments should be performed to better understand the influences of the secretome at the IRSp53 pathway for designing a hopeful platform in most cancer therapies.

## 5. Conclusion

In summary, the present study demonstrated the boosting of *IRSp53* expression in colon cancer specimens. Moreover, we found that hAMSCs secretome is capable of downregulating IRSp53 and EGFR/c-Src/p-AKT/p-Stat3/cyclin D1 signaling pathways in the HT-29 colon cancerous cell line. In fact, the present research supports the notion that the secretome of the MSCs is a novel path in cancer treatment, and IRSp53 can be reckoned as a new key goal in colon cancer remedy.

## Data Availability

This study's data are accessible from the corresponding author upon appropriate demand.
